# A systematic review of the impact of stigma and nihilism on lung cancer outcomes

**DOI:** 10.1186/1471-2407-12-184

**Published:** 2012-05-20

**Authors:** Suzanne K Chambers, Jeffrey Dunn, Stefano Occhipinti, Suzanne Hughes, Peter Baade, Sue Sinclair, Joanne Aitken, Pip Youl, Dianne L O’Connell

**Affiliations:** 1 Griffith Health Institute, Griffith University, Brisbane, Australia; 2 Cancer Council Queensland, Brisbane, Australia; 3 Cancer Council New South Wales, Sydney, Australia; 4 National Lung Cancer Program, Cancer Australia, Sydney, Australia; 5 School of Population Health and Community Medicine, University of New South Wales, Sydney, Australia; 6 School of Medicine and Public Health, University of Newcastle, Newcastle, Australia; 7 Sydney Medical School – Public Health, University of Sydney, Sydney, Australia

**Keywords:** Lung neoplasms, Systematic review, Health-related stigma, Therapeutic nihilism

## Abstract

**Background:**

This study systematically reviewed the evidence on the influence of stigma and nihilism on lung cancer patterns of care; patients’ psychosocial and quality of life (QOL) outcomes; and how this may link to public health programs.

**Methods:**

Medline, EMBASE, ProQuest, CINAHL, PsycINFO databases were searched. Inclusion criteria were: included lung cancer patients and/or partners or caregivers and/or health professionals (either at least 80% of participants had lung cancer or were partners or caregivers of lung cancer patients, or there was a lung cancer specific sub-group focus or analysis), assessed stigma or nihilism with respect to lung cancer and published in English between 1^st^ January 1999 and 31^st^ January 2011. Trial quality and levels of evidence were assessed.

**Results:**

Eighteen articles describing 15 studies met inclusion criteria. The seven qualitative studies were high quality with regard to data collection, analysis and reporting; however most lacked a clear theoretical framework; did not address interviewer bias; or provide a rationale for sample size. The eight quantitative studies were generally of low quality with highly selected samples, non-comparable groups and low participation rates and employed divergent theoretical and measurement approaches. Stigma about lung cancer was reported by patients and health professionals and was related to poorer QOL and higher psychological distress in patients. Clear empirical explorations of nihilism were not evident. There is qualitative evidence that from the patients’ perspectives public health programs contribute to stigma about lung cancer and this was supported by published commentary.

**Conclusions:**

Health-related stigma presents as a part of the lung cancer experience however there are clear limitations in the research to date. Future longitudinal and multi-level research is needed and this should be more clearly linked to relevant theory.

## Background

It is estimated that there were 1.61 million cases of lung cancer diagnosed worldwide in 2008 [1], representing about 12.7% of all new cancers globally. It is the most common cancer among men and the second most common among women [1]. The male:female incidence ratio was approximately 2.1:1, and nearly three-quarters of the cases (71%) were 60 years and over at diagnosis [1]. The highest rates among men were found in Central, Eastern and Southern Europe, Northern America and Eastern Asia, and in Northern America among women [1]. In the more developed countries, incidence rates among males continue to decline, while there is evidence that the increasing rates among females are starting to plateau [2], reflecting previous trends in smoking prevalence. With continuing endemic smoking in many less developed countries, increases in incidence are expected to continue.

Worldwide, contrary to the improved survival outcomes for many other types of cancers, the prognosis for people diagnosed with lung cancer remains poor, with 5-year relative survival being around 6-14% among males and 7–18% among females [2]. Much of this is due to the lack of observable symptoms for early stage lung cancer, meaning that most lung cancers are diagnosed at an advanced stage when treatment options are limited [3-6]. Combined with the high incidence, this poor survival means that lung cancer is the most common cause of cancer-related death worldwide. Due to its high case fatality, lung cancer mortality patterns, including trends over time and international variability, closely resembled those for incidence. Globally there was a male:female lung cancer mortality ratio of 2.2:1 and 75% of lung cancer deaths were among people aged 60 years and over [1].

To date, the key focus of international public health efforts to reduce the lung cancer burden has been to work towards decreasing incidence of the disease through tobacco control [7]. Strategies have included legislation to control the sales and marketing of tobacco products; restrictions on smoking in public spaces; and mass media campaigns to educate the public on the health risks of smoking [8]. These efforts led to dramatic changes in smoking prevalence. In Australia in 1964 male smoking prevalence was 58% and this fell to 21% in 2007, while for women prevalence fell from 28% to 18% [9]. In the United States overall smoking prevalence was 42.4% in 1965 [10] and fell to 19.8% in 2007 [11]. It has been suggested that this public health approach leads to stigmatisation of smokers, and further that stigmatisation of smokers can be viewed as a powerful tool to motivate behaviour change in smokers [12]. The question arises however as to whether this stigmatisation influences the illness experience of people who develop a smoking-related disease.

In this regard it has been proposed that lung cancer patients, more so than those with other cancers, may feel stigmatised by their disease and that this health-related stigma may lead to reluctance to seek treatment as well as having increased feelings of distress about the cancer [13]. Stigma is a complex phenomenon that has been applied to a wide array of contexts and accordingly definitions vary [14]. Stigma as originally defined occurs when society labels someone as tainted and less desirable on the basis of an attribute that marks them out as different [15]. This label connects to a negative stereotype comprising a set of inferred undesirable characteristics that distinguishes the stigmatised class as separate or different to the dominant group. Power is central to the creation of stigma such that stigmatisation cannot occur unless the persons who are labelled as different or deviant feel less powerful than the social group whose views prevail [14].

Stigma is relationship and context-specific where a specific attribute is associated with a negative evaluation that may lead to negative treatment or discrimination and self fulfilling prophecies, stereotype activation, and identity threat [16]. Negative evaluations may be “felt” or “enacted”. A felt negative evaluation is internalised and may lead to shame or guilt associated with having a condition and to the fear of being discriminated against on the grounds of social unacceptability because of that condition. An enacted negative evaluation refers to actual discrimination. Awareness of stigma may influence behaviour in an automatic way amongst those who are stigmatised and others with whom they interact; as well as threatening or harming an individual’s social identity leading to increased stress and poor coping [16].

Supporting this view, stigma has been linked to a broad range of negative outcomes across the domains of mental and physical health, socioeconomic status, and education levels [16]. Health-related stigma may contribute to the burden of illness for both patients and their families through delayed presentation for care, premature termination of treatment, and the amplification of psychological and social morbidity [17,18]. In the case of lung cancer, health-related stigma may be a result of the association between the disease and smoking, the perception of the disease as self-inflicted; its high mortality; and perceptions about the type of death that may be experienced [19,20].

In addition to stigma, it is also proposed that therapeutic nihilism about the treatment of lung cancer may influence patterns of care with regards to patients’ help seeking behaviours; as well as what treatment options health providers will actually offer. Therapeutic nihilism as a concept first arose in the 19^th^ century as a belief that medical science was limited in its ability to treat disease that was considered best left to the healing powers of nature [21]. In more recent times this concept has been applied to the treatment of dementia and mental illness [22]; and lung cancer [23]. Specifically, in the context of lung cancer therapeutic nihilism is defined as the view that medical treatments for this illness are of no value [23]. Commentary suggests that nihilism is a barrier to evidence-based care for lung cancer patients [24,25]. It has also been suggested that lung cancer research is underfunded by both government and community cancer control agencies due to the combined effects of stigma and nihilism and a lack of integration across tobacco control and disease-focussed research [26,27].

The present review aimed to identify and assess current evidence about the influence of stigma and nihilism on outcomes for lung cancer patients including the possible impact of public health programs.

## Methods

As a first step three authors (SC, JD and SO) developed a set of key clinical questions to guide the review. These were grouped according to: medical and treatment outcomes; psychosocial outcomes; and public health program impacts. Before finalisation, these questions were reviewed by a working group that included clinicians, researchers and consumers with experience in lung cancer. The questions conformed to guidelines in which the target population, intervention, comparator, and outcome are clearly stated to guide the review process [28] questions are listed below by key area.

### Key Area 1: Medical and treatment outcomes

• In people with lung cancer are stigma-related negative self-relevant evaluations associated with late presentation for treatment?

• In people with lung cancer are stigma-related negative self-relevant evaluations associated with poor adherence to treatment?

• In people with lung cancer are stigma-related negative self-relevant evaluations associated with poorer survival?

• In people with lung cancer are nihilistic views about the cancer associated with late presentation for treatment?

• In people with lung cancer are nihilistic views about the cancer associated with poor adherence to treatment?

• In people with lung cancer are nihilistic views about the cancer associated with poorer survival?

• In medical professionals are stigma-related negative evaluations about lung cancer patients associated with patterns of treatment?

• In medical professionals are nihilistic views about lung cancer related to patterns of treatment?

### Key Area 2: Psychosocial outcomes

• In people with lung cancer are stigma-related negative self-relevant evaluations associated with lower levels of psychosocial help seeking?

• In people with lung cancer are stigma-related negative self-relevant evaluations associated with greater psychosocial distress?

• In people with lung cancer are stigma-related negative self- relevant evaluations associated with poorer quality of life?

• In people with lung cancer are nihilistic views about the cancer associated with lower levels of psychosocial help seeking?

• In people with lung cancer are nihilistic views about the cancer associated with greater psychosocial distress?

• In people with lung cancer are nihilistic views about the cancer associated with poorer quality of life?

### Key Area 3: Impacts of public health programs

• In people with lung cancer do anti-smoking public health campaigns contribute to stigma-related negative self evaluations?

• In people with lung cancer do anti-smoking public health campaigns contribute to nihilism views about lung cancer?

Next, a systematic review from 1st January 1999 to 31st January 2011 for the key clinical questions was undertaken. Medline (1999 – March Week 4, 2011), EMBASE (1999 – Week 13, 2011), PsycINFO (1999 – March Week 4, 2011), CINAHL (1999 – 28/02/2011) and ProQuest (1999-31/01/2011) databases were searched. The searches contained keywords and subject headings, such as “stigma.mp”, “prejudic$”, “nihilis$.mp.”, “exp Shame/”, “exp Blame/” and “exp Nihilism/”, respectively. These searches were coupled with searches containing keywords and sub-headings aimed at identifying lung cancer-based research such as “exp Lung Neoplasms/”. In addition, the Web of Science database was searched for citations of the landmark paper by Chapple et al., 2004*.* Potentially relevant articles were identified by examining the title and abstract and then retrieved for more detailed evaluation against the inclusion criteria by one reviewer. Their references were reviewed for other potentially relevant articles.

Studies were included if they met the pre-determined inclusion criteria:

• Included lung cancer patients and/or partners or caregivers (either at least 80% of participants had lung cancer or were partners or caregivers of lung cancer patients, or there was a lung cancer specific sub-group analysis) OR included health professionals considering patients with lung cancer;

• Assessed lung cancer specific stigma or nihilism and included an outcome of interest – survival, delayed presentation, treatment adherence or refusal, patterns of care, psychological distress, psychological help seeking or quality of life

OR

Compared stigma or nihilism associated with lung cancer, with stigma or nihilism associated with other cancers

OR

Compared stigma or nihilism experienced by lung cancer patients who had never smoked with stigma or nihilism experienced by those who were former or current smokers

OR

Assessed anti-smoking public health campaigns and the outcomes of lung cancer specific stigma or nihilism;

• Were published in English;

• Were published after 31st December 1998 and prior to 1st February 2011.

Both qualitative and quantitative studies were included. Reviews, editorials, books, dissertations and commentaries were excluded.

The methodological quality of the included studies was assessed independently by two reviewers and differences resolved by consensus with separate criteria for qualitative (SC, SH) and quantitative (SH, DO) studies. The assessment of the quality of qualitative studies is still evolving. Accordingly, a novel assessment form was developed based on criteria held in the literature to denote high quality [29-31]. Criteria included whether: the sampling frame was described, justified, or met; the framework for the study design, methodology and orientation disclosed; interviewer bias was addressed; the method of analysis was described; reliability and validity checks were included; data were clearly presented. To assess the quality of the design of included quantitative cross-sectional studies a tool was adapted from established tools for cohort and case–control studies [32] focussing on representativeness of the study sample (subject selection), selection bias (comparability of groups) and attrition bias (participation rates).

The characteristics and results of the qualitative and quantitative studies were summarised in tables by one reviewer and then checked by a second reviewer.

## Results

### Search results

**Figure 1:**
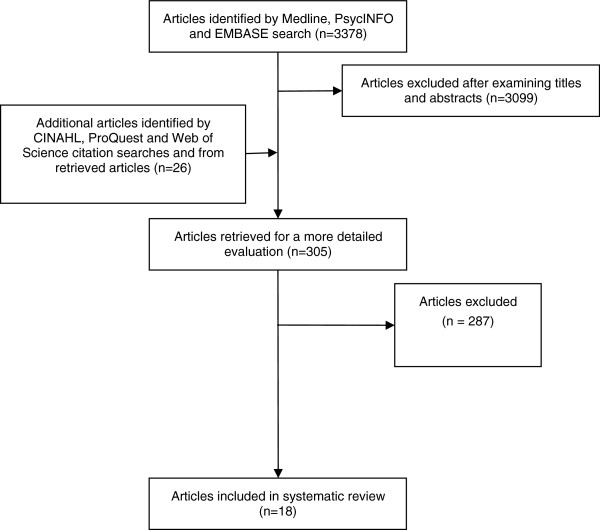
Final process of inclusion and exclusion of studies for the literature review.

The process of identifying relevant articles for the review is outlined in Figure 1. The combined Medline, EMBASE and PsycINFO database search identified 3378 citations. On examination of titles and abstracts, 279 were considered potentially relevant. The CINAHL, ProQuest and Web of Science Citation searches identified another 7, 3 and 2 potentially relevant citations respectively. Another 14 potentially relevant citations were identified from retrieved articles. In total, 305 potentially relevant articles were retrieved. Of these, eighteen articles met the inclusion criteria for the review: 9 articles described 7 qualitative studies and 9 articles described 8 quantitative studies. Of the quantitative studies, seven were cross-sectional and one was a cohort study but only the baseline cross-sectional data were relevant to this review. Of the 287 excluded articles most did not assess stigma or nihilism specifically associated with lung cancer.

### Study quality

The characteristics of the included studies are displayed in Tables 1, 2, and 3. The methodological quality of the included studies is summarised in Tables 4 and 5. Most qualitative studies provided a rationale for sample selection, but a clear rationale for sample size was less common. Only one study [33] provided a qualitative framework and interviewer bias was only addressed in two studies [34,35]. However, use of objective methods for data collection was uniform; most studies included some checks for data credibility; and data presentation was clear in all studies. The qualitative studies were all undertaken with participants in the United Kingdom or North America. Based on the criteria devised by Daly et al. 2007 [30], six were level III studies and one was level IV with data presented for only one lung cancer patient [35].

**Table 1:** Characteristics and results of qualitative studies on stigma and nihilism in lung cancer

**Study**	**Design (Level of evidence)**	**Participants**	**Aim of interview**	**Study factor**	**Results**
Chapple 2004a & b, UK	Home interview (Level III)	Lung cancer patients	Lung cancer patients’ experience of lung cancer including their perceptions, how others reacted to the diagnosis and financial issues	Stigma	Some participants perceived lung cancer as being viewed in the broader society as a self-inflicted disease resulting from smoking and leading to a horrible death. One participant noted that the stigma applied to all lung cancer patients; smokers and non-smokers. As a result of the smoking related stigma it was thought that lung cancer research and screening was neglected.
		N = 45			The press was criticised for blaming lung cancer patients in particular for their disease.
		NSCLC, SCLC and mesothelioma; All stages.			*Medical and treatment outcomes*
		Recruited through general practices, nurses, oncologists, chest physicians and support groups and through study website.			Smoking related stigma was thought to be a reason for lung cancer symptoms not being taken as seriously as those for other cancers leading to delays in diagnosis.
					*Psychosocial outcomes*
					Stigma was perceived to result in social isolation, and deterred support group participation (1 participant) and seeking financial relief (1 participant).
Conlon 2010, USA	Interview, (Level III)	Oncology social workers	Social workers’ perceptions of the lung cancer experience	Stigma	*Smoking stigma*
		N = 18			Lung cancer was always associated with smoking and patients often reported stigma, guilt, blame and shame. Smoking stigma was seen as a reason why support, funding and advocacy for lung cancer were lower.
		Recruited from 17 cancer hospitals in 13 states with experience with approximately 25,000 lung cancer patients.			Division between lung cancer patient smokers and non-smokers.
					*Poor prognosis stigma*
					Patient reported lung cancer stigmatised as being mostly fatal.
					*Psychosocial outcomes*
					Patients reported smoking stigma sometimes resulted in reluctance to tell others that they have lung cancer.
					*Psychosocial outcomes*
					Poor prognosis stigma potentially led to difficulties attending support groups.
Corner 2005 & 2006, UK	Semi-structured interviews with a time-line prompt mostly in home and often with a relative present (Level III)	Patients recently (<3 months) diagnosed with lung cancer	To explore delays in lung cancer diagnosis	Stigma	*Medical and treatment outcomes*
		All experienced symptoms for 4 months or more prior to visiting doctor			Factors potentially leading to delay in seeking medical treatment included expectation and fear that smokers would be denied treatment (reported by 1 participant who was a smoker)
		N = 22; 12 men, 10 women			
		Median age = 68 years			
		15/22 inoperable disease			
		1/22 never smoker			
		Recruited from 2 hospital outpatient clinics.			
Leydon 2003, UK	Telephone and face-to-face semi-structured interviews (Level IV)	Cancer patients diagnosed < 2 years ago with a focus on those of lower SES	Perceptions of cancer diagnostic process	Lung cancer specific fear	*Medical and treatment outcomes*
		N = 17; 5 men, 12 women			Lung cancer viewed as fatal (by 1 participant). This theme was reported as arising in the context of potential barriers to seeing a doctor
		Included 2 lung cancer patients; a 67 year old male and a 59 year old female.			
		Recruited through cancer support community organisations.			
Sharf 2005 USA, Texas	Interview with guiding questions (Level III)	Patients with NSCLC or a suspicious pulmonary mass who refused or did not follow-up for physician-recommended treatment (N = 7) or invasive investigation (N = 2).	Reasons for declining physician-recommended treatment or follow–up options	Nihilism	*Medical and treatment outcomes*
		100% male, 89% white			Reasons reported included the view that lung cancer treatments were futile (5 participants).
		Identified at multidisciplinary pulmonary conferences and review of pathology reports at a university affiliated Veterans Affairs hospital.			
		9/31 eligible patients interviewed			
		2 with history of depression			
Tod 2008, UK	Semi-structured home interviews with partner or a friend participating at the request of 12 participants (Level III)	Lung cancer patients	Factors influencing delay in reporting symptoms (patient delay)	Stigma	*Medical and treatment outcomes*
		N = 20; 12 men, 8 women.		Nihilism	Factors identified that might result in patient delay in consulting a doctor about their symptoms included the stigma that it was caused by smoking and fear.
		18 diagnosed in past 6 months			
		3 non smokers; 9 previous smokers.			
		Recruited from deprived health district by a respiratory physician and lung cancer nurse specialists.			
Tod 2010 UK	3 focus groups, (Level III)	Focus group 1; 6 community pharmacists (50% female)	Factors influencing delay in reporting symptoms (patient delay)	Stigma	*Medical and treatment outcomes*
		Focus group 2: 6 clinical nurse specialists (100% female)			Factors identified that might result in lung cancer patient delay in consulting a doctor about their symptoms included fear of negative evaluation and expectation of denial of treatment especially for smokers.
		Focus group 3: 2 practice nurses (100% female)			
		Recruited an area with high levels of lung cancer and smoking and a history of heavy industry			

*NSCLC* = Non small cell lung cancer; *SCLC* = Small cell lung cancer.

**Table 2:** Table of characteristics of included quantitative studies of patients’ perceptions and caregivers’ attitudes

**Study**	**Design (Level of evidence)**	**Participants**	**Study factors/Patient groups**	**Outcomes**	**Comments and quality**
LoConte 2008: Else-Quest 2009, Wisconsin USA	Cohort	NSCLC, breast or prostate cancer	Lung cancer (N = 96) vs breast cancer	Guilt and shame (SSGS)	Primary endpoint = SSGS
	Mailed patient self report survey (Level IV as only cross-sectional baseline data were relevant)	Stage IV	(N = 30) or prostate cancer	Perceived cancer related stigma	Target sample size lung cancer
		Fluent and able to complete survey in English	(N = 46)	Perceived stigma	N = 94, breast cancer N = 47, prostate cancer N = 47 to detect anticipated difference of > 0.75 points in mean SSGS scores with 80% power for a 2-sided significance level of 0.05
		Recruited from 3 oncology clinics			Study closed prematurely because of poor accrual among breast cancer patients
		Mean age, years (SD)			
		Lung cancer = 65.6 (11)			
		Breast cancer = 61.8 (9.8)			
		Prostate cancer = 72.9 (9.2)			
		200/237 recruited			
		172/200 (86%) completed at least 1 questionnaire			
	Cross sectional				*Study quality*
	Mailed patient self report survey (Level IV)	Lung cancer patients	Current or former smokers (N = 88) vs never smokers	Guilt and shame	Subject selection 0
		(n = 96)	(N = 8)	Perceived cancer	Group comparability 0
		49% women	Perceived stigma	related stigma	Participation rate 0
			Guilt and shame	Anxiety	
				Anger	
				Depression	
				Self esteem	
Cataldo 2011, USA	Cross sectional	Lung cancer all types and stages	Lung cancer stigma	Depression	Outcomes used to validate lung cancer stigma scale
	Patient self report online survey (Level IV)	Convenience sample		Self esteem	
		Recruited via websites frequented by potential study participants		Social support	
		70% female		Social conflict	*Study quality*
		21% never smoked		Quality of life	Subject selection 0
		Mean age, years (SD) = 55 (13.7)			Group comparability NA
		186/200 completed all stigma items			Participation rate 0
Devitt 2010, Victoria, Australia	Cross sectional		Shame about lung cancer as a potential barrier to participating in a support group		12% of participants reported attending a face-to-face support group
	Patient self report survey (Level IV)	Lung cancer (74% NSCLC, 16% SCLC, 5% mesothelioma, 5% presumed lung cancer)			53% of participants indicated they would be likely or very likely to attend a support group for lung cancer patients
		42% Stage IV			Also surveyed support group facilitators
		Able to complete survey in English			
		Consecutive lung cancer patients attending multidisciplinary outpatient clinics at a cancer centre subsequent to initial consultation			*Study quality*
		Excluded those with cognitive impairment or ECOG performance status > 2			Subject selection 0
		12% current smokers			Group comparability NA
		Median age, years = 68			Participation rate 0
		42% female			
		Response rate = 101/172 (59%)			
Lobchuk 2008b, Canada	Cross sectional	Primary caregivers of lung cancer patients (76% NSCLC)	Primary caregiver blame re patient’s efforts to control the disease	Primary caregiver assistance in coping with lung cancer and its symptoms	
	Preliminary sample	58% diagnosed with advanced disease			*Study quality*
	Primary caregiver self report survey (Level IV)	Able to speak, read and write in English and cognitively competent			Subject selection 0
		Convenience sample recruited from 5 outpatient cancer clinics			Group comparability 0
		Patients	current (N = 25) vs former (N = 66) vs never (N = 9) smokers	Primary caregiver blame re patient’s efforts to control the disease	Participation rate 0
		9% never smokers			
		Mean age, years (SD) = 64 (8.0)			
		62% female			
		Response rate = 100/350 (29%)			
Siminoff 2010, USA, Ohio	Cross sectional	Lung cancer patients with a primary caregiver	Family blames the cancer on the patient for not taking better care of themselves	Patient depression	
	Patient and their primary caregiver semi- structured interview, (Level IV)	Stage III or IV NSCLC	Patient and caregiver perceptions		*Study quality*
		Recruited from a comprehensive cancer centre and its community affiliates – identified by their physicians			Subject selection 0
		92% smokers			Group comparability 1
		Mean age, years (SD) = 65 (9.7)			(adjusted for age and sex)
		45% female			Participation rate 0
		Response rate = 76%			
		N = 190 patients + caregivers			

*ECOG* = Eastern Co-operative Oncology Group; *NSCLC* = Non small cell lung cancer; *SCLC* = Small cell lung cancer; *SSGS* = State Shame and Guilt Scale; *NA* = Not applicable (only within individual correlations were reported so comparability of groups was not assessed).

**Table 3:** Table of characteristics of included quantitative studies of health professionals’ perceptions of lung cancer

**Study**	**Design (Level of evidence)**	**Health professionals**	**Study factors/Patient groups**	**Outcomes**	**Comments and quality**
Jennens 2004, Australia	Cross sectional	All Australian general, pulmonary and palliative care physicians, medical and radiation oncologists and thoracic surgeons (N = 1325) who saw at least one patient a year with metastatic lung cancer	Pessimism regarding the use of platinum based chemotherapy for stage IV NSCLC	Referrals for chemotherapy for stage IV NSCLC	Referrals to chemotherapy is included as part of the measure of pessimism
	Mailed self report survey (Level IV)	N = 544			*Study quality*
		Response rate = 51%			Subject selection 2
					Group comparability NA
					Participation rate 0
Schroen 2000, USA	Cross sectional	Members of American College of Chest Physicians self reportedly practising either pulmonary medicine or thoracic surgery and treating adult lung cancer patients	Nihilism – underestimation of survival rate for resected stage I NSCLC	Beliefs re survival benefit for chemotherapy for various stages of NSCLC and radiotherapy for resected disease	Considered gender, treatment volumes, date of medical training completion
	Mailed self report survey (Level IV)	Randomly selected			Thoracic surgeons and pulmonologists see patients early in their diagnosis and refer patients to medical and radiation oncologists
		Pulmonologists N = 594 (response rate = 50%)			*Study quality*
		Thoracic surgeons N = 416 (response rate = 52%)			Subject selection 0
					Group comparability 0
					Participation rate 0
Wassenaar 2007, Wisconsin USA	Cross sectional	All 1132 members of the American college of Physicians- Internal Medicine or the American College of Family Physicians in Wisconsin	Lung (NSCLC) vs breast cancer	Referrals to clinical oncologist	Physicians answering lung cancer questionnaire saw average 4.12 lung cancer patients/year.
	Mailed self report survey (Level IV)	Randomly allocated scenarios with lung or breast cancer patients, smokers or non smokers at stage 1B, M1 and end of life		Beliefs re survival benefits of chemotherapy for various cancer stages	Anticipated response rate at least 30%
		N = 672			Sample size chosen to detect differences of at least 25% in the response patterns between disease groups with 80% power for a two-sided significance level of 5%
		Response rate = 59.4%			*Study quality*
					Subject selection 1
					Group comparability 2
					Participation rate 0

*NSCLC* = Non small cell lung cancer: *NA* = Not applicable (only within individual correlations were reported so comparability of groups was not assessed).

**Table 4:** Methodological quality of included qualitative studies (n = 7)

**Quality category**	**Studies meeting criterion, n (%)**
**1. Sample**	
**(a) Clear and/or Justified Sampling frame**	
2. Clear rationale for sample selection	5 (71)
1.Convenience sample (e.g., volunteers)	2 (29)
0. Sampling rationale not described and/or clear	0
**(b) Adequacy of sample size**	
2. Rationale for sample size provided and met	3 (43)
1. Rationale for sample size provided but not met	0
0. No rationale provided	4 (57)
**(c) Adequacy of sample description**	
2. Sample adequately described	3 (43)
1. Sample partially described	4 (57)
0. Sample not described	0
**2. Qualitative framework (theoretical orientation e.g., feminism, interpretivism, critical theory)**	
2. Framework provided for study design and methodology and orientation disclosed	1 (14)
0. No framework provided	6 (86)
**3. Interviewer bias addressed**	
2.Yes	2 (29)
0. No	5 (71)
**4. Data recording**	
2. Objective methods used for data capture (e.g., tape recording, transcription)	7 (100)
0. Subjective methods used or methods not described	0
**5. Data analysis**	
2. Method of analysis described (e.g., thematic analysis, interpretative, phenomenological analysis, content analysis) and detailed	5 (71)
1. Either method of analysis described only or detailed only	2 (29)
0. Method of analysis not described or detailed	0
**6. Reliability and validity**	
2. Checks for data credibility are provided (e.g., triangulation, audits and continual recoding, intercoder and intracoder reliability)	3 (43)
1.Partial checks for data credibility	2 (29)
0. No clear checks provided for reliability and validity of qualitative approach	2 (29)
**7. Data presentation**	
2. Examples of data presented that provide an understanding of data analysis and interpretation (one or two quotes or specific examples)	7 (100)
1. Examples provided but do not present a clear interpretation of data	0
0. Very little data presented	0

**Table 5:** Methodological quality of included quantitative studies (n = 8)

**Quality category**	**Studies meeting criterion, n (%)**
**1. Subject Selection**	
2. Representative of population of interest	1 (12.5)
1. Selected group	1 (12.5)
0. Highly selected or not described	6 (75.0)
**2. Comparability of groups analysed on demographic characteristics**	
2. Comparable (or matched)	1 (12.5)
1. Not comparable but adjusted analysis used	1 (12.5)
0. Not comparable and not adjusted for differences	3 (37.5)
Not applicable: no comparisons made	3 (37.5)
**3. Participation rate**	
2. High participation rate (>80%) and no important differences between participants and non-participants	0
1. Moderate participation rate (65-80%) and no important difference between participants and non-participants	0
0. Low participation rate (<65%), important differences between participants and non- participants or not described	8 (100)

All of the quantitative studies provided cross-sectional data (level IV evidence) using divergent theoretical and measurement approaches (Tables 2 and 3). All but two of the studies [36,37] used samples from highly selected populations, limiting the generalisability of the findings. Three studies reported correlations between measures on the same individuals [36,38,39] and of the remaining five studies, only one compared outcomes in groups that were comparable on important potential confounding factors [37]. All had low participation rates with important differences between participants and non-participants or did not report whether there were important differences.

### Lung cancer related stigma

The results from the qualitative studies are summarised in Table 1; and quantitative results are summarised in Tables 6, 7, and 8. The qualitative studies identified health-related stigma as part of the experience of lung cancer. Patients reported feeling stigmatised by the prevailing view that if someone had lung cancer they would necessarily be a smoker and have inflicted this disease on themselves; and this view was seen by patients as unfair [20,40]. Patients feared that they would be denied treatment and thought that lung cancer was neglected in research and screening because of the link between smoking and lung cancer [20,41,42]. Social workers working with lung cancer patients reported very similar themes when discussing their perceptions of the lung cancer experience [34]. It was proposed that the association of lung cancer with smoking led to lung cancer patients feeling stigmatised, from which guilt, blame and shame arises. This stigma was internalised by patients and led to a division amongst lung cancer patients between smokers who ‘deserve their cancer’ and non-smokers who do not. The view that lung cancer is mostly fatal was also described as another form of stigma.

**Table 6:** Results of quantitative studies comparing different patient groups

**Study**	**Participants**	**Outcome**	**Main findings**
Lung (N = 96) vs breast (N = 30) or prostate (N = 46) cancer patients			
LoConte 2008: Else-Quest 2009 USA			*Baseline differences between groups*
	Stage IV	*Patient*	
	Lung cancer patients	Guilt and shame (SSGS)	NS^
	100% NSCLC	Shame subscale	NS^
		Perceived cancer related stigma (5 items)	p < 0.01^ greater for those with lung cancer
Lung (N = 89) vs breast (N = 30) vs prostate (N = 43) cancer patients			
LoConte 2008: Else-Quest 2009 USA			*Baseline differences between groups*
	Stage IV	*Patient*	
	Lung cancer patients	Guilt and shame (SSGS)	NS^^
	100% NSCLC	Perceived stigma (single item)	NS^^
Smoking history (N = 88) vs Never smoker (N = 8) lung cancer patients			
LoConte 2008: Else-Quest 2009 USA			*Differences between groups*
	Stage IV NSCLC	*Patient*	
		Guilt and shame (SSGS)	p = 0.02* greater for those with a smoking history
		Perceived cancer related stigma (5 items)	NR
Current smoker (N = 25) vs Former smoker (N = 66) vs Never smoker (N = 9) lung cancer patients			
Lobchuk 2008b Canada	58% diagnosed with	*Patient*	*Differences between groups*
	advanced disease 76% NSCLC	Primary caregiver blame re patient’s efforts to control the disease - single item	p < 0.05^^ greater for current vs never smokers
			p < 0.05^^ greater for former vs never smokers
Lung vs breast cancer			
Wassenaar 2007 USA		*Physician*	*Differences between groups*
	Different stages	Referrals to clinical oncologist for the scenarios:	
		after surgery for stage 1B disease	p = 0.86*
		hepatic and lung metastases – good performance status	p < 0.001* lower for lung cancer
		metastases - poor performance status	p < 0.001* lower for lung cancer
		advanced disease – solely for supportive or palliative care	p = 0.009* higher for lung cancer
		Reported importance of type of cancer as a factor contributing to decision to refer to oncologist	p = 0.19^#^
		Belief that chemotherapy could improve survival for:	
		stage IB resected disease	p = 0.001* lower for lung cancer
		metastatic disease – good performance status	p = 0.015* lower for lung cancer

*ECOG* = Eastern Co-operative Oncology Group; *NSCLC* = Non small cell lung cancer; *SCLC* = Small cell lung cancer; *SSGS* = State Shame and Guilt Scale; *NR* = Not reported; *NS* = Not statistically significantly different; ^ MANCOVA with sex, age and time since diagnosis taken into account; ^^ Univariate ANOVA; * 2-sided *t* test: * Chi-squared or Fisher’s exact test: ^#^ Non parametric Wilcoxon rank sum test.

**Table 7:** Results of quantitative studies examining effects of stigma-related negative evaluations on psychosocial outcomes

**Study**	**Participants**	**Study Factor(s)**	**Outcome**	**Main findings**
LoConte 2008: Else-Quest 2009, USA	Stage IV NSCLC			*Association between stigma or self blame and outcomes*
		Perceived stigma		
		(1 item)	Self esteem (RSES)	NS**
			Direct effect	p< 0.01^#^ Negative association
			Indirect effects via self-blame (SSGS)	
			Anxiety (State-Trait Anxiety Inventory)	p< 0.01**Positive association
			Direct effect	p< 0.05^#^ Positive association
			Indirect effects via self-blame (SSGS)	
			Anger (State-Trait Anger Inventory)	p< 0.01** Positive association
			Direct effect	p< 0.01^#^ Positive association
			Indirect effects via self-blame (SSGS)	
			Depression (shortened CES-D)	p< 0.01** Positive association
			Direct effect	p< 0.01^#^ Positive association
			Indirect effects via self-blame (SSGS)	
		Self Blame (SSGS) adjusted for perceived stigma	Self esteem (RSES)	p< 0.01** Negative association
			Anxiety (State-Trait Anxiety Inventory)	p< 0.01** Positive association
			Anger (State-Trait Anger Inventory)	p< 0.01** Positive association
			Depression (shortened CES-D)	p< 0.01** Positive association
Cataldo 2011, USA	All types and stages of lung cancer			
		Lung cancer stigma scale (Cataldo scale - 43 items)	Depression (CES-D)	p< 0.01* Positive association
			Quality of life (Quality of Life Inventory)	p< 0.01* Negative association
			Self esteem (RSES)	p< 0.01* Negative association
			Social support (Social Support Indices)	
			Availability	p< 0.01* Negative association
			Validation	p< 0.01* Negative association
			Subjective social integration (Social Support Indices)	p< 0.01* Negative association
			Social conflict (Social Support Indices)	p< 0.01* Positive association
		Lung cancer stigma scale Stigma and shame subscale (19 items)	Depression (CES-D)	p< 0.01* Positive association
			Quality of life (Quality of Life Inventory)	p< 0.01* Negative association
			Self esteem (RSES)	p< 0.01* Negative association
			Social support (Social Support Indices)	
			Availability	p< 0.01* Negative association
			Validation	p< 0.01* Negative association
			Subjective social integration (Social Support Indices)	p< 0.01* Negative association
			Social conflict (Social Support Indices)	p< 0.01* Positive association
Devitt 2010, Victoria, Australia	42% Stage IV 74% NSCLC	Shame about lung cancer	Participation in a support group	10% of patients reported shame as a potential barrier
				29% of support group facilitators thought patients’ shame was a potential barrier
Lobchuk 2008b, Canada	Primary caregivers of lung cancer patients			*Correlation between caregiver blame and caregiver assistance*
	58% advanced disease	Primary caregiver blame re patient’s efforts to control the disease (single item)	Primary caregiver assistance in coping with lung cancer and its symptoms (single item)	r = 0.044, p = 0.66
	76% NSCLC			
Siminoff 2010, USA, Ohio	Stage III or IV NSCLC	Family blames cancer on the patient		*Regression coefficient for blame and depression*
		Patient agrees	Patient Depression (CES-D)	
		Familial cohesion		p< 0.05^1^ Positive association
		Familial expressiveness		p< 0.05^2^ Positive association
		Familial conflict		p< 0.05^3^ Positive association
		Caregiver agrees	Patient Depression (CES-D)	
		Familial cohesion		p< 0.05^1^ Positive association
		Familial expressiveness		p< 0.05^2^ Positive association
		Familial conflict		p< 0.05^3^ Positive association

*ECOG* = Eastern Co-operative Oncology Group; *NSCLC* = Non small cell lung cancer; *SCLC* = Small cell lung cancer; *SSGS* = State Shame and Guilt Scale; *CES-D* = Center for Epidemiological Studies Depression Scale; *NA* = Not applicable; NS = Not statistically significantly different; *RSES* = Rosenberg’s Self-Esteem Scale; r = correlation coefficient; * Two-sided test; ** Multiple regression analyses; ^#^ bootstrapping; ^1^ Multi-level model including age, gender, physical health, relationship of caregiver to patient, familial cohesion; ^2^ Multi-level model including age, gender, physical health, relationship of caregiver to patient, familial expressiveness; ^3^ Multi-level model including age, gender, physical health, relationship of caregiver to patient, familial.

**Table 8:** Results of quantitative studies examining effects of nihilistic views of health professionals on medical and treatment outcomes

**Study**	**Health professionals**	**Outcome**	**Main findings**
**Pessimism** regarding the use of platinum based chemotherapy for stage IV NSCLC			
Jennens 2004 Australia	Physicians, medical and radiation oncologists and thoracic surgeons who saw patients with metastatic lung cancer	**Referrals for chemotherapy** for stage IV NSCLC	Does not examine the effect of pessimism on referrals - the outcome of interest, referrals for chemotherapy, is included as part of the measure of pessimism
**Pessimists** vs **realists** vs **optimists** (underestimation vs realistic estimation vs overestimation of survival rate for resected stage I NSCLC)			
Schroen 2000 USA	Pulmonologists and thoracic surgeons treating adult lung cancer patients		*Differences between pessimists, realists and optimists*
		**Believe in survival benefit in NSCLC for chemotherapy**:	
		As adjuvant for resected stage I-IIIA disease	p = 0.07*
		In addition to radiotherapy for unresectable locally advanced disease	p < 0.001* lower for pessimists
		For stage IV disease	p = 0.31*
		**Believe in palliative benefit for chemotherapy** for stage IV NSCLC	p = 0.19*
		**Believe in survival benefit for adjuvant radiotherapy** in resected stage I-IIIA NSCLC	p = 0.66*

*NSCLC* = Non small cell lung cancer; *Chi-squared test.

Two quantitative studies examined the level of stigma and stigma related outcomes such as blame in different patient groups (Table 6). Perceived stigma was greater for lung cancer patients and guilt, shame and blame were greater for those lung cancer patients who had a history of smoking. In a study of patients with advanced cancer, lung cancer patients reported more perceived cancer-related stigma (measured by a five item instrument) compared to breast and prostate cancer patients but there was no significant difference in the levels of perceived stigma (measured by a single item) between lung, breast and prostate cancer patients [43]. While levels of guilt and shame were not significantly higher for lung cancer patients compared with breast or prostate cancer (individually or combined), within the lung cancer cohort current or former smokers had higher guilt and shame than never smokers. The second study [44] found that caregiver blame regarding the patients’ efforts to control the disease was greater for patients who were current or former smokers compared with never smokers.

### Stigma and medical and treatment outcomes

There were no studies evaluating the possible effects of stigma-related negative evaluations on adherence to treatment, survival or patterns of care. There were no quantitative studies examining the effects of stigma-related negative evaluations on late presentation. Four qualitative studies identified smoking related stigma as a possible reason for late presentation (Table 1). In Chapple 2004, a patient with mesothelioma felt that smoking associated stigma resulted in lung cancer symptoms not being taken seriously which then resulted in delays in diagnosis [20]. In Corner 2005 a patient reported an expectation and social view that treatment for lung disease would likely be denied to smokers, and this was given as a reason for delay in seeking medical treatment for symptoms [41,42]. Tod et al. (2008) [40] reported that some patients with lung cancer including non smokers expected to be stigmatised and to be blamed for their disease and so delayed seeking medical help for their symptoms. Health professionals also reported stigma as a reason for lung cancer patients not seeking medical care for symptoms, on the basis of fear that treatment would be denied and fear of negative evaluation [45].

### Stigma and psychosocial outcomes

Support group attendance was the only psychosocial help seeking outcome addressed in the literature. Two qualitative studies reported that the stigma associated with lung cancer could lead to difficulties in attending support groups (Table 1) [20,34]. In a survey of recently diagnosed lung cancer patients 10% reported shame would be a potential barrier to support group attendance (Table 7) [39].

The effect of stigma-related negative evaluation on psychological distress was assessed in three quantitative studies (Table 7). Cataldo (2011) [38] followed the approach of Berger at al (2001) [46] to develop a lung cancer-specific measure of health-related stigma. The components considered were precursors (e.g., knowledge about societal attitudes to smoking); perceived stigma (e.g., social isolation, discrimination and shame); and individual responses (e.g., emotional symptoms, behavioural responses). The authors found that higher depression, lower self-esteem, lower social support, poorer social integration and higher social conflict were associated with greater lung cancer specific stigma. Consistent with this, Siminoff et al. (2010) [47] found that stage III and IV lung cancer patients’ depression was greater when they believed that their family blamed them for their cancer. Else-Quest et al., 2009 [48] applied attribution theory and the ‘looking-glass’ model and found that self-blame mediated the link between perceived stigma and adjustment outcomes in lung, breast and prostate cancer patients. However, differences were found in the mediational pathways between cancer patient groups. Perceived stigma and self-blame explained more of the variance in self-esteem, anger, anxiety, and depressed affect in lung cancer patients compared to patients with breast or prostate cancer. Lung cancer patients were also more likely to indicate that their own behaviour contributed to their cancer. In a group of patients with advanced lung cancer anxiety, anger and depression were associated with perceived stigma (measured by a single item). These associations were partly explained by self-blame which was also associated with increased anger, depression and anxiety [48].

In two qualitative studies lung cancer patients and oncology social workers reported that the perception of lung cancer as a self-inflicted disease that leads to a horrible death results in social isolation (Table 1) [20,34]. A lung cancer patient also reported being reluctant to seek financial help as a result of shame, however in the quotes reported it was not clear that this in itself was linked to the type of cancer [49].

Two quantitative studies (Table 7) examined the effects of stigma or related outcomes on quality of life. One found that poorer quality of life was associated with greater lung cancer specific stigma [38]. The other study applied the theory of social motivation, justice and moral emotions (2000) [50] to assess how illness attribution reactions influence caregiver behaviour and found that primary caregiver blame was not associated with their reported assistance to the patient in coping with lung cancer and its symptoms [44].

### Lung cancer related nihilism

Qualitative studies reported that lung cancer was seen by patients as a fatal disease (Table 1) [34,35,40]. No empirical studies directly addressed therapeutic nihilism, which may reflect difficulties in operationalising and measuring this construct. Consistent with this, no instruments purporting to directly measure therapeutic nihilism were identified. There was some indirect evidence of greater therapeutic nihilism with respect to lung cancer in the primary care setting (Table 6) [37]. A single quantitative study examining physicians’ referral preferences in response to various scenarios found that physicians were less likely to refer advanced lung cancer patients to an oncologist compared to patients with advanced breast cancer except for supportive or palliative care, and they were less likely to believe in the survival benefits of chemotherapy for resected stage Ib or metastatic lung cancer. It was proposed that this may at least in part be due to nihilism about lung cancer as a disease with a poor prognosis that warrants a less aggressive treatment approach [37].

### Nihilism and medical and treatment outcomes

There were no studies evaluating the possible effects of patients’ nihilistic views about lung cancer on survival. No quantitative studies examined the effect of patients’ nihilistic views about lung cancer on delays in presentation or treatment adherence. Table 1 shows the results for the three qualitative studies that identified patient nihilism as a possible factor influencing medical and treatment outcomes. Two qualitative studies identified nihilism as a possible reason for patients’ delays in seeking medical treatment for their symptoms [35,40] and a third qualitative study found that one of the reasons why patients refused recommended treatments and investigations for lung cancer was that they believed treatment was futile [33].

Two quantitative studies approached the question of therapeutic nihilism by measuring beliefs regarding a survival benefit for chemotherapy (Table 8). Schroen [51] used the underestimation of 5-year survival for Stage I disease as an indicator of pessimism about treatment. In this study those categorised as pessimistic were significantly less likely to believe in a survival benefit for chemotherapy in addition to radiotherapy for unresectable locally advanced non small cell lung cancer (NSCLC). An Australian study [36] found a wide variation in knowledge about chemotherapy for metastatic lung cancer and concluded that pulmonary physicians, radiation oncologists and palliative care physicians most often rated chemotherapy as not useful in this setting compared to medical oncologists. However this study did not examine the effect of pessimism on referrals for chemotherapy, rather chemotherapy referral for stage IV NSCLC was one of the items that contributed to the measure of pessimism.

### Nihilism and psychosocial outcomes

No studies were found that evaluated the possible effects of patient or medical practitioner nihilism on psychosocial outcomes.

### Impact of public health anti-smoking programs

This review focused on the views of patients and of health professionals. While authors of several of the qualitative studies raised the issue of the effect of anti-smoking public health campaigns on patient or health professional stigma and nihilism [20,34,40-42,45], no studies were found that directly examined how or whether anti-smoking campaigns impact on patient or health professional stigma related negative self evaluations or nihilistic views about lung cancer. Qualitative studies did however provide some insights. Lung cancer patients reported that tobacco control advertisements were distressing to watch and that the press reinforced the smoking related stigma [20]. Tod et al., 2008 [40] noted that patients saw information campaigns as contributing “to fatalistic views as they focused on death rather than treatment.”

## Discussion

This review suggests that health-related stigma is part of the lung cancer experience. Specifically, patients felt that negative social views about lung cancer being a self-inflicted disease with a mostly fatal outcome meant that treatment might be delayed or denied; and that seeking treatment was futile [20,40-42]. Stigma appears to be experienced more by lung cancer patients than other patient groups; and more by smokers compared to non-smokers [43]. Qualitative research with health professionals mirrored these views [34,45]. Hence, it seems reasonable to conclude, despite the limitations of the research to date with respect to quality and levels of evidence, that stigma is an important issue in the case of people with lung cancer and those close to them.

It is unclear however the extent to which this ‘felt’ health-related stigma actually influences medical and treatment outcomes. Quantitative studies with physicians found evidence of pessimistic views about referral to specialist oncology care for lung cancer patients and an underestimation of survival, with effects greater for some physician sub groups [36,37,51]. However, these studies addressed the issues of stigma and nihilism indirectly. Hence, while it is plausible that these factors do have a negative effect on medical and treatment outcomes for lung cancer patients with regards to presentation for and adherence to treatment, it is not possible on the basis of research to date to confirm or quantify such an effect. Further, the determinants of survival outcomes are likely to be multifactorial including factors such as socioeconomic status and rurality, as well as disease features. Stigma-related negative self-evaluations by lung cancer patients were associated with higher psychological distress and poorer quality of life [38,46,48]. Stigma appears to be internalised as shame, guilt and blame; and the influence of perceived stigma and self blame on outcomes seems to be strong for lung cancer patients [48]. From this it does appear that health-related stigma has a uniquely negative effect on psychosocial outcomes for lung cancer patients.

Therapeutic nihilism was addressed in these studies in an indirect way, through physician self-report of referral or treatment approaches for lung cancer patients. Classical definitions of therapeutic nihilism incorporate beliefs about medical science being limited and potentially harmful; and illness best left to nature [21]. The modern version of the Hippocratic oath includes the statement: “I will apply, for the benefit of the sick, all measures [that] are required, avoiding those twin traps of overtreatment and therapeutic nihilism” [52]. Future research to better operationalise and measure therapeutic nihilism in current times appears warranted and this could usefully include antecedents of this belief as well as therapeutic outcomes.

**Figure 2:**
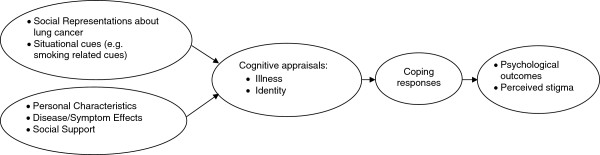
Proposed Model for the influence of stigma on lung cancer outcomes.

Limitations in the research with regard to study design, sampling frames and low participation rates were noted. These limitations may be related to a number of factors. First, people with lung cancer are often unwell at the time of diagnosis, or become unwell soon after. This presents challenges in recruitment, assessment, and study retention and this remains an ongoing challenge for researchers in this field [53]. Second, the studies identified did not use consistent theoretical frameworks and the consequent variations in assessment approaches make it difficult to draw strong conclusions from the available evidence. One potential approach would be to incorporate stigma into a broader model of adjustment to cancer, such as transactional models of stress and coping [54,55]. This approach has been previously suggested as a framework for explaining stigma-related identity threat and takes into account collective representations, situational cues, and personal characteristics as precursors that influence threat appraisal from which individual responses and outcomes evolve [16]. Applying this to stigma and cancer links social representations about lung cancer (stigma); situational cues (e.g., anti-tobacco advertisements or smoking-related cues); and personal characteristics and coping resources (e.g., disease stage, optimism, social support) to consider how these shape that person’s cognitive appraisals of the threat the disease poses to their health and future; and their identity. These appraisals then in turn shape the person’s coping responses and influence their psychological outcomes (see Figure 2). This framework may have efficacy in guiding future descriptive research in this area as well as the design of psychosocial interventions.

It is of note that one study followed a clear and systematic approach to develop a measure of health-related stigma [38]. Specifically, an expert multidisciplinary panel was recruited to modify an existing stigma scale [46] to be relevant to the experience of people with lung cancer, after which an online survey was conducted to confirm the factor structure and check the criterion-related validity and internal consistency of the scale. However, further research is needed to fully establish the construct validity of this scale and this should include evidence of discriminant and predictive validity as well as cross-cultural applicability. While a cancer site-specific stigma scale is difficult to use for comparisons across different cancer types, a benefit is that a more specific scale will tap into the unique aspects of stigma that are associated with lung cancer.

With regards to the impact of public health programs on stigma-related negative self-evaluation in lung cancer patients, qualitative data suggest that media advertisements depicting smoking-related illness may contribute to patients’ distress [20,40]. While data here are sparse, it does seem that a raised social awareness of lung cancer as *necessarily* smoking-related has contributed to stigma-related negative self-perceptions for lung cancer patients. Commentary on this matter has included consideration of the weighing up of the public benefit that ensues from the decrease in tobacco-related disease when smoking prevalence rates decrease against the potential cost to those who are stigmatised [12,56,57]. Clearly this is a complex matter, however social or community education activities to combat health-related stigma in lung cancer may be needed. In this regard, efforts to reduce health-related stigma in lung cancer will necessarily need to be multilevel. While the impact of stigma on people with lung cancer may be individual and clinical in nature (e.g., increased distress or decreased quality of life), the phenomenon is social in nature. Specifically, stigma arises out of a social context where a characteristic or attribute of a class of people leads them to be negatively stereotyped with consequential disadvantage and compromised outcomes [14,16]. Hence, the social context also needs to be addressed alongside efforts to reduce negative individual sequelae of stigma.

## Conclusion

In conclusion, this review suggests that health-related stigma is part of the lung cancer experience; and that it contributes to psychological distress for patients and impairs quality of life. Therapeutic nihilism appears to, at least in part, be embedded in the experience of stigma. How stigma and nihilsm may influence health professional behaviour is unclear. It seems clear that there are deficits in health professionals’ knowledge about contemporary evidence-based lung cancer care and this needs to be addressed. Moreover, longitudinal research to examine the relative influence of individual level variables (e.g., stigma-related negative self-perceptions); and group level factors (e.g., socio-economic and geographic variables) is needed to clearly identify targets for change. Commentary suggests tobacco control activities may be linked to health-related stigma in lung cancer however this is a complex issue with little clear empirical data on the topic [25,58-60]. More broadly, it has been suggested that there needs to be a dialogue between tobacco control researchers and lung cancer care researchers and clinicians to develop an integrated approach to lung cancer research, policy and services planning [27]. This is an area for future action by health policy makers, health care professionals, and consumer advocates.

## Competing interests

There are no competing interests for this paper.

## Authors’ contributions

SC and DO’C led the design of the study, undertook data analysis and drafted the manuscript. SH undertook data analysis and helped to draft the manuscript. JD, SO, PB, SS and PY participated in the design of the study and helped to draft the manuscript. All authors read and approved the final manuscript.

## Pre-publication history

The pre-publication history for this paper can be accessed here:

http://www.biomedcentral.com/1471-2407/12/184/prepub
